# Insights into Nanomedicine for Immunotherapeutics in Squamous Cell Carcinoma of the head and neck

**DOI:** 10.7150/ijbs.47068

**Published:** 2020-07-19

**Authors:** Qiang Xu, Meiyu Fang, Jing Zhu, Haoru Dong, Jun Cao, Lin Yan, Fransisca Leonard, Felix Oppel, Holger Sudhoff, Andreas M. Kaufmann, Andreas E. Albers, Xu Qian

**Affiliations:** 1 Cancer Hospital of the University of Chinese Academy of Sciences (Zhejiang Cancer Hospital); Institute of Cancer and Basic Medicine (IBMC), Chinese Academy of Sciences. Hangzhou, P.R. China.; 2 Key Laboratory of Head & Neck Cancer Translational Research of Zhejiang Province, Cancer Hospital of the University of Chinese Academy of Sciences (Zhejiang Cancer Hospital); Institute of Cancer and Basic Medicine (IBMC), Chinese Academy of Sciences. Hangzhou, P.R. China.; 3 Department of Clinical Laboratory, Cancer Hospital of the University of Chinese Academy of Sciences (Zhejiang Cancer Hospital); Institute of Cancer and Basic Medicine (IBMC), Chinese Academy of Sciences. Hangzhou, P.R. China.; 4 First School of Clinical Medicine, Wenzhou Medical University, Wenzhou, P.R. China.; 5 Department of Neurosurgery, Xuanwu Hospital, Capital Medical University, Beijing, P.R. China.; 6 Department of Nanomedicine, Houston Methodist Research Institute, Houston, USA.; 7 Department of Otolaryngology, Head and Neck Surgery, Klinikum Bielefeld, Bielefeld, Germany.; 8 Clinic for Gynecology, Charité-Universitätsmedizin Berlin, corporate member of Freie Universität Berlin, Humboldt-Universität zu Berlin, and Berlin Institute of Health, Berlin, Germany.; 9 Department of Otolaryngology, Head and Neck Surgery, Charité-Universitätsmedizin Berlin, corporate member of Freie Universität Berlin, Humboldt-Universität zu Berlin, and Berlin Institute of Health, Berlin, Germany.

**Keywords:** nanotherapeutics, drug delivery, cancer immunotherapy, nanovaccine, head and neck squamous cell carcinoma, human papillomavirus

## Abstract

Immunotherapies such as immune checkpoint blockade benefit only a portion of patients with head and neck squamous cell carcinoma. The multidisciplinary field of nanomedicine is emerging as a promising strategy to achieve maximal anti-tumor effect in cancer immunotherapy and to turn non-responders into responders. Various methods have been developed to deliver therapeutic agents that can overcome bio-barriers, improve therapeutic delivery into the tumor and lymphoid tissues and reduce adverse effects in normal tissues. Additional modification strategies also have been employed to improve targeting and boost cytotoxic T cell-based immune responses. Here, we review the state-of-the-art use of nanotechnologies in the laboratory, in advanced preclinical phases as well as those running through clinical trials assessing their advantages and challenges.

## Introduction

Head and neck squamous cell carcinoma (HNSCC) still remains the sixth most common malignancy worldwide with >830,000 cases and >430,000 deaths each year 1. Although the overall 5-year survival rate of patients with HNSCC is about 40-60%, in cases with locoregionally advanced disease this number may be much lower 2. During the past few years, cancer immunotherapy, especially immune checkpoint blockade (ICB) approaches, were able to generate durable immune responses and led to improved survival in patients with advanced HNSCC, according to the results of clinical trials 3, 4. In 2019, pembrolizumab, an anti-programmed cell death-1 (anti-PD1) antibody, in combination with chemotherapy has been approved for the first-line treatment of patients with recurrent or distant metastatic (R/M) HNSCC. However, the overall response rates were about 20% in advanced HNSCC patients who received PD1 or PD-ligand (L)1 checkpoint inhibitor treatments 4-6. Notably, a number of severe immune-related adverse events such as dermatitis, colitis, hepatitis, and pneumonitis are also developed in some patients and required a delayed administration of ICB treatment or other interventions 7, 8. Other strategies like adoptive cell transfer or anti-cancer vaccines are limited by reduced T cell activity, the development of autoimmune toxicity or weak immunogenicity 9, 10. Studies revealed that the heterogeneity in the spatial distribution of tumor-infiltrating lymphocytes (TILs), cancer stem cell (CSCs)-related immune invasion and the immunosuppressive microenvironment are the main factors contributing to the lower efficacy of ICB treatment at clinical stage 10, 11. In addition, similarly to the conventional cytotoxic chemotherapy drugs, immunotherapy is constrained by transport processes. The limited capability of immune-checkpoint inhibitors to permeate into the targeted solid tumor tissue would compromise the treatment efficacy 12, 13. Thus, a conceptual understanding of cancer immunotherapy resistance as well as the development and implementation of pipelines to maximize therapeutic outcomes and reduce severe immune-related systemic toxicity is urgently needed.

One important consideration for the development of optimized immunotherapies is nanomedicine, i.e. utilizing nanotechnology to improve the transport of therapeutics selectively into tumor tissue, remodel the immunity, minimize toxicity and immune-related adverse events 14. First, nanosized carriers can incorporate several functional elements to protect drugs from degradation, improve sustained drug release, enhance permeation, and deliver tumor-associated antigens (TAA), chemotherapy drugs, phototherapy sensitizers, siRNAs, etc. 13. These characteristics would enable the achievement of therapeutic concentrations of drugs with limited toxicity. Second, the ability to recognize pathological tissues distinct from normal tissues would improve site-specific delivery of nanotherapeutics 13. One example is the multistage vector platform (MSV), composed of three components with different manipulation that can display different biodistribution profiles 15. The first stage vector is porous silicon microparticles which can circulate in the blood and overcome transport bio-barriers to reach the tumor vasculature. The second stage vector is nanoparticles (NPs) loaded in the pores of porous silicon microparticles that can be released into the tissue upon the degradation of silicon materials. Finally, therapeutic small molecules loaded in the nanoparticles can be delivered and effective on both tumor cells and immune cells 15. Taking advantages of these unique properties, preclinical studies have demonstrated that these approaches are successful in cancer vaccination by co-delivery of TAAs, neoantigens or adjuvants to dendritic cells (DCs), the design of artificial antigen presenting cells (aAPCs) and reversing the immunosuppressive microenvironment 14.

In this review, we summarize nanotechnology based strategies in the development of treatment modalities to improve the outcome and to decrease the toxicity of immunotherapeutics against HNSCCs. Ultimately, such efforts will help to find the potential targets and build a foundation for the development of novel nano-immunotherapeutics for HNSCC.

## Immuno-oncology features of HNSCC

HNSCC is an immunosuppressive disease with phenotypic and functional intratumor cellular heterogeneity 16, 17. HNSCC tumors interfere with the immune system by employing many mechanisms that modulate functions of immune cells to develop immune evasion and immune escape (for more detailed information, please refer to reviews by Albers et al. and Qian et al. 18, 19). The potential mechanisms for the dysfunction of biological steps in immunity against cancer cells being responsible for the limited response to ICB treatment have been explored in HNSCC 11, 20-26. Recently, three tumor-immunophenotypes and related molecular pathways have been recognized according to the spatial distribution of T cells within the tumor microenvironment 23. The presence of high PD-L1 expression, high density of TILs and B cells, IFN-γ signatures and intact antigen presentation (i.e., high tumor mutation burden) is characterized as the inflamed phenotype 23, 27. Immune-excluded phenotype is defined as an immunosuppressive tumor microenvironment by the presence of myeloid-derived suppressor cells (MDSCs), reactive stroma, TGF-β signatures, low major histocompatibility complex class I (MHC-I) expression, low TILs and angiogenesis 16, 27, 28. Immune-desert tumors are associated with lack of TILs, neuroendocrine features, low MHC-I expression, fatty acid metabolism and Wnt/β-catenin signaling 23, 27. A clinical study has further identified distinct immunological profiles in oropharyngeal cancer (OPC) being related to ICB responses 26. In addition, activation of alterative immune checkpoints such as T-cell immunoglobulin mucin-3 (TIM-3) were identified to confer resistance to therapeutic PD-1 inhibition 29. Moreover, we and others found that CSCs play an important role in immune suppression, immune evasion and immune escape. The non-specific target of HNSCC-CSCs may contribute to treatment resistance, tumor recurrence and metastasis 10, 16. The immune heterogeneity described herein raises clinical challenges for patient stratification and for designing a well-tailored immunotherapy.

Currently, carcinogen-exposure-associated tumors represent the majority of HNSCCs with a rising incidence of high-risk human papillomavirus (HR-HPV)-driven HNSCCs 30. HR-HPV-driven HNSCCs show distinct clinical presentations regarding treatment response, overall survival and prognosis compared to HR-HPV negative HNSCCs 31. Accordingly, the 8th edition of the American Joint Committee on Cancer (AJCC) downstaged the HR-HPV-associated OPC compared to HR-HPV negative OPC 32. Notably, the immune features of HR-HPV-driven tumors are distinguishable from HR-HPV negative tumors. For example, a B-cell associated signature within the population of TILs was prominent in HR-HPV positive HNSCCs compared to HPV-negative HNSCCs 33. A spectrum of different immune lineages between HR-HPV-driven and -negative HNSCC has recently been demonstrated by a single-cell RNA sequencing (scRNA-seq) analysis 34. Specifically, CD4^+^ T follicular helper (CD4^+^ Tconv) cells (TFP) were significantly enriched in TILs of HR-HPV-driven tumors. HNSCC patients with a gene expression signature associated with CD4^+^ Tconv cells had a longer progression-free survival 34. The identified differences between the two tumor types within the same anatomical site may exist due to virus-derived immunity. Notably, TFP play a dominant role in the production of long-lasting humoral immunity which is important for immunization 35. Further, differentiation of CD4^+^ T cells into either TFP or type 1 helper T (TH1) cells can be modulated by exposure of DCs to type I interferon in a recombinant vesicular stomatitis virus (VSV) infection model 36. Regarding to the ICB treatment, patients with HR-HPV^+^ HNSCC had a better response to anti-PD-1 treatment 37. The total PD-1^+^ TIL was identified to be higher in HR-HPV^+^ patients with better clinical outcome 38. RNA-sequencing analysis demonstrates that patients with HR-HPV^+^ OPC has immune rich phenotype and were associated with a favorable response to anti-PD-1/PD-L1 therapy 26. Further, dysfunctional PD-1^high^ CD8^+^ TILs were more frequent in HR-HPV negative patients with worse outcome 38. However, nivolumab treatment produces tumor regression in only a minority of patients with recurrent HR-HPV-driven cancer 37. A recent study demonstrated that treatment with nivolumab in addition with the HR-HPV 16 vaccine ISA101 increased the overall response rate to 33% comparing to 16% to 22% with ICB alone in patients with HR-HPV-driven tumor 39. Ultimately, a substantial strategy for immunotherapy in HNSCC and an in-depth study of the dissimilar of immune responses between the two tumor types would be necessary.

## Nanotechnology-based-drug-delivery systems

Nano-scaled materials such as liposomes, micelles, MSV and metal-organic frameworks (MOFs) are optimized as nanocarriers to incorporate with anti-cancer drugs or biomolecules for the development of localized drug delivery systems 40. For example, anticancer drugs including doxorubicin, paclitaxel, cisplatin, topotecan, 5-fluorouracil and small interfering RNA (siRNA) have been developed for cancer nanotherapeutics 41, 42. In HNSCCs, earlier studies demonstrated that tumor growth was inhibited and survival was prolonged in response to treatment with cisplatin-loaded polymeric micelles (CDDP/m) in mouse models 12, 43. Specifically, the delayed release of CDDP from the polymeric shell can overcome the degradation of free drug by interaction with glutathione 12. More importantly, this platform is able to concurrently target CSCs, because CSCs have an increased glutathione level compared to bulk tumor cells. Additionally, greater therapeutic efficacy was achieved upon utilization of cyclic Arg-Gly-Asp (cRGD) peptide-installed CDDP/m (cRGD-CDDP/m) that the αvβ5 integrins overexpressed in HNSCC-CSCs could be successfully recognized by the cRGD peptide 44. Moreover, cRGD-CDDP/m has been found to home to lymph node metastases rapidly 44. In this regard, clinical trials are currently underway to test the feasibility of cRGD-CDDP/m against pancreatic cancer (NC-6004, Nanocarrier Co., Ltd) and are expected to be investigated in HNSCC. Similar results have been achieved with nanoparticles formulated with 5-FU or Doxorubicin *in vitro* and *in vivo* compared to free drugs 45, 46. Notably, the MSV platform incorporating three components on different spatial scales (microparticles, nanoparticles and small molecules) is designed to overcome a wide range of biological barriers through bio-nano interactions 15. At clinical stage, Abraxane, nanoparticle albumin-bound paclitaxel has been evaluated as a part of combination chemo-radiotherapy in locally advanced HNSCC and R/M HNSCC 47.

**Figure 1:**
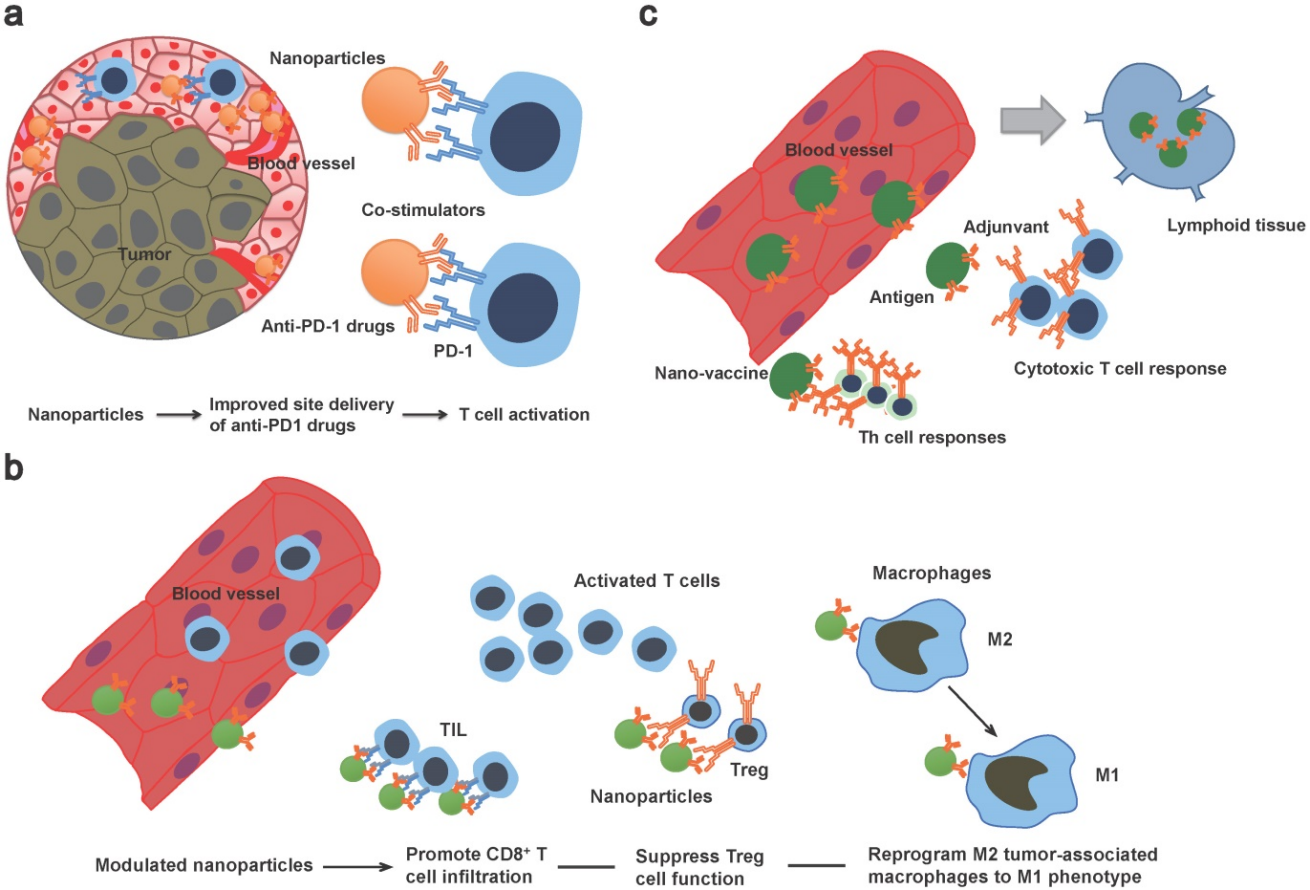
Schematic illustration of multifunctional properties of nanoimmunotherapeutics. a) Nanotechnology-based theranostic approaches can improve transport spatiotemporally. Co-delivery of stimulators or conventional drugs can be developed as combination therapy. b) Modulated nanoplatforms can prime a suppressive tumor microenvironment. c) Nanovaccine co-delivered tumor antigens and adjuvants can be drained into lymphoid tissue and induce strong antigen specific cytotoxic T cell and Th cell responses. TIL: tumor-infiltrating lymphocytes.

In addition to nanoformulated conventional drugs, modulation of immune checkpoint inhibitors is also expected to improve the treatment efficacy and overcome sequential immune-related side effects (Figure 1, Table 1). For instance, a design of co-delivery of anti-PD1 and antitumor necrosis factor receptor superfamily member 4 (aOX40) by PLGA nanoparticles can spatiotemporally co-delivery drugs into the tumor site 48. Specifically, higher rates of T cell activation and increased immunological memory with enhanced therapeutic efficacy were observed in melanoma and breast tumor models 48. This dual immunotherapy nanoparticle-based platform demonstrates a novel strategy to improve the combination immunotherapy. Another strategy is utilizing nanomaterials that enable triggered activation or induce drug release in pathological tissue specifically. A clinical stage example is drug CX-072, a protease-cleavable Probody therapeutic directed against programmed cell death ligand 1 (PD-L1), for patients with advanced or recurrent solid tumors or lymphomas that currently is in phase I/II clinical trials (https://clinicaltrials.gov/ct2/show/NCT03013491). In particular, the antigen-binding site of the Probodies is masked with a peptide, and in the tumor microenvironment, the masking peptide can be cleaved by tumor-associated proteases that enable the release of Probody antibody 49. This approach can minimize the antigen binding to normal cells and reduce autoimmune-like effects 49.

## Immune-priming approaches

In order to maximize the immune therapeutic effect, modulating the tumor microenvironment is also under investigation (Figure 1). For example, an *in vivo* T cell targeted drug delivery system incorporating nanoparticles with antibodies and small molecules has been developed. Compared to free drugs, this approach enables less dosage to confer the ICB effect of PD1^+^ T cells and to reduce toxicity 50. Additionally, this approach co-delivers a TLR7/8 agonist that can promote CD8^+^ T cell infiltration into the tumor site 50. In another study, tLyp1 peptide-modified hybrid nanoparticles conjugated with the drug Imatinib was shown to target and modulate intratumoral Treg cell suppression through inhibition of STAT3 and STAT5 phosphorylation 51. When combined with anti-cytotoxic T-lymphocyte antigen-4 (anti-CTLA-4) treatment, it was shown to reduce of Treg cells and increase of CD8+ T cells infiltration at the tumor site and consequently elevate the survival rate in a mouse model 51. Zhang *et al*. developed lipid nanoparticles incorporating with the tumor-targeting peptide iRGD, a PI3K inhibitor and a α-GalCer agonist of therapeutic T cells 52. They found this systematic treatment could reverse the tumor microenvironment from immune-suppressive to immune-stimulatory and enable tumor-specific CAR-T cells homing to the cancer lesion 52. In addition to modulating the T cells, nanoparticles formulated with mRNAs encoding interferon regulatory factor 5 and IKKb kinase have shown its ability to reprogram tumor-associated macrophages from M2 phenotype to M1 phenotype. Because M2 phenotype is associated with immunosuppressive functions leading to tumor progression, metastases and therapy resistance, this complement approach would enable physicians to obviate immunosuppression and to design a companion strategy to augment the treatment efficacy of immunotherapy 53. It should be noted that, a recent study demonstrated that maintaining both phenotypes of M1 and M2 in the tumor microenvironment with a fine-turned ratio rather than a polarization to all-M1 population would improve the treatment efficacy of nanoparticle-mediated chemotherapy 54. Taken together, these findings present a straightforward solution to improve antitumor effect of immunotherapy by a repertoire of nanoparticles loaded with drugs in modulating the tumor microenvironment.

## Nanovaccines

Successful therapeutic cancer vaccines depend on the recognition of tumor specific antigens, co-delivery of adjuvant and the delivery vehicles. In HNSCC, specific antigens such as HR-HPV oncogenic proteins, p53 and CSC-related proteins can prime immune cells to induce a robust immune response 19, 55. For example, vaccination targeted to HR-HPV E6 and E7 oncoproteins can induce T-cell responses against HPV-16 and a complete histologic response 39, 56. Adjuvant DC-based vaccination against p53 has shown modest vaccine-specific immunity in patients with HNSCC 55. Other strategies, e.g. targeting stem cell transcription factors like NANOG might eradicate CSCs particularly 57. However, their clinical efficacies may be limited in advanced HNSCC due to the immunosuppressive factors in the tumor microenvironment and by the sequential physical spatio-temporal peculiarities 19. Nanomedicine has the potential to facilitate vaccination-induced antitumor effects and to reverse immune suppression. Because therapeutic agents can be freely selected and congregated on nanocarriers according to the intended application, nanovaccine can not only codeliver tumor antigens and adjuvants to lymphoid tissues in close proximity, but also further enhance therapeutic efficacy by loading with immunosuppressive inhibitors or immunostimulatory compounds 58.

**Figure 2:**
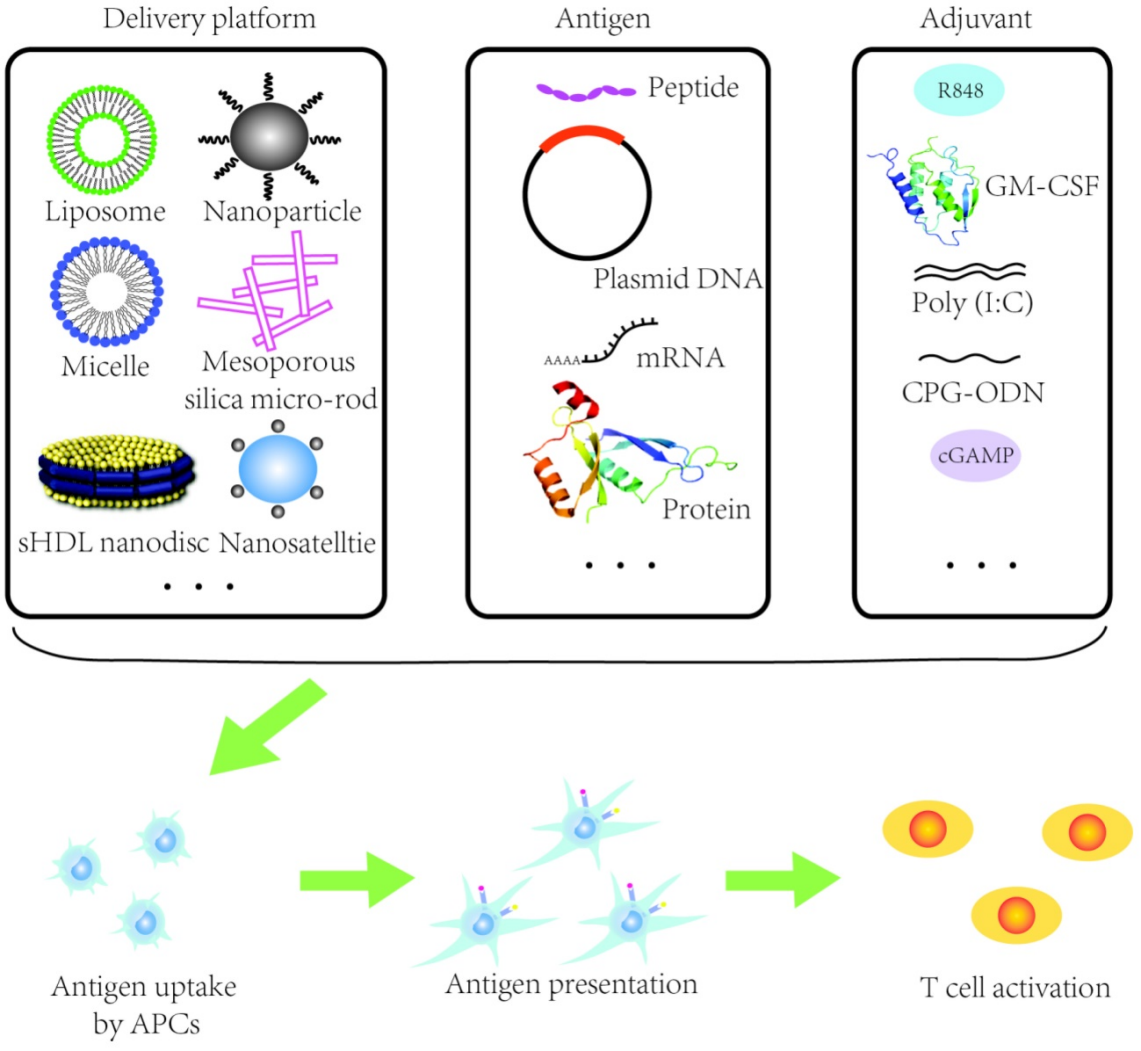
Schematic illustration of a nanovaccine. APCs: antigen presenting cells. sHDL: synthetic high-density lipoprotein. Ploy(I:C): Ploy-deoxy-inosinic-deoxy-cytidylic acid. GM-CSF: granulocyte-macrophage colony stimulating factor. CpG-ODN: CpG oligodeoxynucleotides. cGAMP: Cyclic guanosine monophosphate-adenosine monophosphate.

"Proof of concept" studies have shown that nanocarriers can successfully co-deliver tumor antigens and adjuvants and the expansion of tumor-specific T cells can be rapidly potentiated (Figure 2) 59. Nanomaterials such as liposomes or poly lactic-co-glycolic acid (PLGA) nanoparticles have been utilized to design therapeutic HR-HPV vaccine (Table 1). It has been shown that HR-HPV nanovaccines induce a strongly T cell immunity in preclinical studies. For instance, a liposomal HPV16 mRNA formulation, RNA-lipoplex (RNA-LPX) was administered intravenously in murine HR-HPV16-positive TC-1 and C3 tumor models 60. This approach displayed a robust E7 antigen-specific CD8^+^ T cell response with a strong and sustainable memory phenotype and a less immune suppressive microenvironment 60. Moreover, the combination of anti-PD-L1 treatment augmented the complete remissions of tumor and improved overall survival, and importantly, a late tumor relapse 60. Nanovaccine with tumor antigen MUC1 mRNA designed against triple negative breast cancer in combination with an anti-CTLA-4 monoclonal antibody can be successfully delivered to DCs in lymph nodes resulting in an enhanced T cell anti-tumor immune response 61. MUC1 is expressed in HNSCC and it has been shown in a MUC1-specific CAR-T cell therapy approach *in vitro* and *in vivo* 62. In another approach, researchers fused cancer cells with DCs and used the fusion cells' membrane to derive cytomembrane nanovaccine particles that simulated a direct T cell activation and indirect DC-mediated T cell activation 63. The therapeutic effect of nanovaccines was conferred by their phenotype mimicking tumor cells and antigen presenting cells simultaneously resulting in strong immune responses against cancer cells. Despite breast and colorectal cancer being used in this study, DC-HNSCC cell fusions have been created before 64 and could be employed similarly in future studies.

As shown above, nanovaccines hold a great promise as a tool to synergize with ICB. Recently, Tan *et al*. identified the oncogene SOX2 to facilitate the immune suppression by the STING-type I interferon (IFN-I) signaling pathway in HNSCCs 65. The authors further developed a novel nanosatellite vaccine delivery system incorporating STING agonist and tumor antigens and demonstrated a rapid accumulation in the lymph node, improved IFN-I signaling and TIL in the tumor microenvironment 65. Moreover, a combination of nanosatellite vaccine with anti-PD-L1 significantly expands tumor-specific CTLs and reduces PD-1^high^ and Tim3^+^ CD8^+^ CTL 65. PC7A NP, a synthetic polymeric nanoparticle that incorporates tumor antigens can induce strong antigen-specific cytotoxic T cell and Th cell responses in tumor models of melanoma, colon cancer, and HPV-E6/E7 TC-1 tumors 66. Notably, PC7A NPPC7A NP is an ultra-pH sensitive nanoparticle that can transport into draining lymph nodes successfully and potentiate efficient cytosolic delivery of tumor antigens to antigen presenting cells (APCs) where type I interferon-stimulated genes were activated 66. Moreover, a long-term antitumor memory could be induced by the PC7A NP treatment, as seen by an inhibition of tumor formation in those tumor-free mice that were previously treated with nanovaccine and then rechallenged with tumor cells in the HPV-E6/E7 TC-1 tumor model 66. Synergistic with anti-PD1 antibodies, the PC7A nanovaccine has also shown an improved anti-tumor immunity and survival in the TC-1 model 66. In line with these studies, a HR-HPV nanovaccine formulating the CL 1,2-dioleoyl-3-trimethyl-ammonium-propane (DOTAP) and long HR-HPV peptides can successfully boost strong anti-tumor immunity and synergize with an anti-PD1 checkpoint inhibitor 67. As illustrated in Table 1, these studies have been focused on current vaccine strategies. More targets are currently discovered and nanomedicine is constantly evolving, promising a better impact for future immunotherapeutics.

A clinical study has shown that a liposomal vaccine combined with idiotype, a tumor-specific antigen, and adjuvant interleukin-2 (IL-2) induces sustained tumor-specific T-cell responses in lymphoma patients 68. It has been demonstrated that nanoparticles can also protect the degradation of mRNA vaccines to improve the systemic delivery of antigens to DCs where *de novo* T cell responses against vaccine antigens were observed *in vitro* and in patients with advanced melanoma in a phase I clinical trial 69. Further, a multifunctional RNA-loaded magnetic liposomes (RNA-NPs) platform was developed to initiate potent antitumor immunity and importantly, to predict the responders after vaccination with magnetic resonance imaging (MRI) 70. RNA-NPs incorporated with the T2 MRI contrast-enhancing effects of iron oxide nanoparticles can enhance DCs transfection and detect DCs migration to lymph nodes with MRI. These effects have been seen in 2 days after vaccination and the reductions of tumors were correlated with survival in murine B16F10-OVA tumor models. Recently, a combinatorial design of biodegradable polymeric DNA nanoparticles for local delivery in solid tumors has been developed 71. This platform utilizes nonviral cargo poly (beta-amino ester)s (PBAE)-based nanoparticles to deliver DNA to tumor cells expressing MHC-I and ultimately, induces expression of the co-stimulatory molecule 4-1BBL and IL-12 secretion which leads to activation of cell-mediated cytotoxic immune responses. These genetically reprogrammed tumor cells are therefore termed tumor-associated antigen-presenting cells. This approach can avoid the intrinsic immunogenicity or toxicity as commonly seen in vectors like viruses or lipid nanoparticles 71. Additionally, various nanoparticles have been utilized for developing therapeutic T cells for adoptive therapies. Please refer to a review by Yang et al. for more detailed information 59.

## Combination therapy

Application of nanomedicine to develop combination therapy has been studied to improve the median survival with long-term memory responses in cancer patients who receive immunotherapy. In the clinical setting, only a fraction of patients display immune response to antitumor immunotherapy 10. Toxicities associated with ICB are a pivotal issue where the adjustment of drug ratios to optimize clinical efficacy and safety are considered 72. In addition, several studies demonstrated that treatment failure is mainly related to immune suppressive factors in the tumor microenvironment 73. Given of the ability of nanocarriers to load various anticancer drugs, it is important to consider the combination of more than one therapeutic using nanotechnology, which is a significant advantage to increase the clinical response of cancer immunotherapy. One consideration is utilizing nanocarriers to combine chemotherapeutic drugs to trigger immunogenic cell death (ICD) of cancer cells, a process that can induce immune activation. For example, combinations of doxorubicin and anti-PD1 drugs can be delivered by synthetic high-density lipoprotein (sHDL) nanodiscs which can not only reduce the off-target toxicity of chemo-drugs, but also amplify antitumor CD8^+^ T cell responses 74. This may be important because as seen in a recent study, there were no significant changes of posttreatment to pretreatment median CD8+TIL density ratio in OPC patients who received durvalumab (PD-L1 inhibitor) ordurvalumab plus tremelimumab (CTLA-4 inhibitor) 75. Yang *et al*. developed a nanovesicle platform which includes pH-responsive nanovesicles (pRNVs) self-assembled from block copolymer polyethylene glycol-b-cationic polypeptide (PEG-b-cPPT) 76. These nanovesicles can encapsulate a photosensitizer and indoximod, an indoleamine 2, 3-dioxygenase inhibitor, to improve efficient drug delivery. The dual combination can also induce reactive oxygen species (ROS) generation and ICD effects. Importantly, the recruitment of DCs was increased, the immune response was activated and the tumor microenvironment was modulated via indoximod with increased CD8^+^ T cell infiltration 76. Other approaches such as immunotherapy combined with photothermal therapy or photodynamic therapy or gene therapy were also reported (for a detailed overview, please refer to a review by Nam et al.) 77. Remarkably, these strategies not only improve the synthetic site-specific drug delivery but also induce robust immune activation.

## Conclusions and prospections

Taking advantage of the physiochemical properties of various nanomaterials, nanotechnology-based theranostic approaches have been explored to improve transport and biodistribution of therapeutic drugs, reduce side effects and consequently, widen the therapeutic window in curative cancer treatments and other diseases 78, 79. To date, clinically available nanodrugs for cancer treatment such as Abraxance (albumin-bound paclitaxel) and Vyxeos (lioposomal daunorubicin and cytarabine) are applied in R/M HNSCC, breast, lung, and pancreatic cancer, metastatic breast cancer and high-risk acute myeloid leukemia 47, 80, 81. In addition, preclinical studies have shown that HNSCC-CSCs can be targeted through multidisciplinary nanotechnology-based approaches. Specifically, strategies to prime the tumor microenvironment in cases of recurrent or metastatic HNSCC have the potential to improve immune therapeutic outcomes. Preclinical study and clinical trials have shown the promising results of combination therapy with immunotherapy and conventional therapy 39. Thus, the therapeutic development of HNSCC can be achieved as a result of the enormous progress in nanomedicine.

Although the concept of nanoimmunotherapeutics is not too far from clinical reality, there are a number of obstacles that needed to be addressed to move preclinical findings to clinical application. For example, the pharmacokinetics, stability and circulation time of nanoimmunotherapeutics in the bloodstream, the effectiveness and the long-term safety of the delivery systems should be further investigated and the feasibility of these platforms in functional preclinical HNSCC models is necessary to advance for clinical trials. However, remodeling the tumor microenvironment remains challenge to establish in the preclinical models. Given by the heterogeneity within tumor and between individuals as discussed in previous sections, a multi-modal approach that combines engineering strategy and multiple therapeutics should be explored to augment the efficacy of immunotherapies and to improve site-specific delivery. One example is the image-guided therapeutic where nanoparticle-based immunotherapeutic platforms in combination with MRI have been employed successfully to achieve local and systemic anti-tumor effects 70. In addition, patient stratification and biomarkers that can be exploited to predict immunotherapy response are also of importance to tailor treatment. Importantly, beyond the advantage of drug-delivery systems, nanomedicine would also improve the understanding of the underlying mechanisms and contribute to cancer diagnostics 54. Notably, a recent study identified clonotypes of T cells are related to clinical responses to ICB by analyzing deep single-cell sequencing of RNA and T cell receptor repertoires in tumor tissue, adjacent normal tissue and blood. These clonotypes can also be detected in peripheral blood of responsive patients which may promote the development of rational biomarkers 82. However, it remains largely unknown for immune-excluded and immune-desert tumors.

Taken together, innovation in nanotechnology design will likely synergize the current practical approach to elicit robust treatment response and to build a deeper quantitative and conceptual understanding of cancer disease in the era of immunotherapy.

## Figures and Tables

**Table 1:** Examples of nanoimmunotherapeutics in head and neck squamous cell carcinoma

Delivery platform	Composition	Cancer model	References
**Immunotherapy**			
Multidomain peptide assembled nanofibrous matrix	K2(SL)6K2 multidomain peptide, Cyclic dinucleotides	Mouse HNSCC (MOC2-E6E7 cells)	83
PC7A nanoparticle	2-(Hexamethyleneimino) ethyl methacrylate (C7A-MA) monomer, PEG-b-PC7A copolymer, HPV-16 E7 peptide	TC-1 tumor-bearing mice	66
PLGA nanoparticle	Poly (lactic-co-glycolic acid), Nano-diamino-tetrac	Oral cancer cell lines (OEC-M1 cells)	84
Tocopherol-modified hyaluronic acid nano suspension	Tocopherol-modified hyaluronic acid (HA-Toco), TLR7/8 dual agonist resiquimod (R848)	Mouse OSCC (AT84 cells)	85
Combination therapy			
Nanoscale metal-organic framework	DBP-Hf nMOF based on 5,15-di (p-benzoato) porphyrin bridging ligand, Indoleamine 2,3-dioxygenase	Mouse HNSCC (SQ20B cells)	86
Polydopamine coated spiky gold nanoparticles	Polydopamine coated spiky gold nanoparticles, Doxorubicin	Mouse HNSCC, lung metastasis (TC-1 cells)	87
Liposome	DOTMA: cholesterol 1: 1, Murine Interleukin 2, Murine Interleukin 12 plasmid	Mouse HNSCC (SCCVII cells)	88
Co-assembled binary telodendrimers nanoparticle	PEG5K-CA4-ICGD4 (PCI): a linear PEG block, four dendritic hydrophobic photothermal conversion agents (indocyanine green derivatives, ICGD) and four dendritic cholic acids (CA); PEG5K-Cys4-L8-CA8 (PCLC): PEG, four cysteines and eight CA. PCI: PCLC 1: 1, Doxorubicin, Imiquimod	Mouse oral cancer (OSC-3 cells)	89
Polyanhydride nanoparticle (20: 80 CPTEG: CPH)	20: 80 CPTEG: CPH. CPTEG: 1,8-bis (p-carboxyphenox-y)-3,6-dioxaoctane; CPH: 1,6-bis (p-carboxyphenoxy) hexane, IL-1α	Mouse HNSCC (SQ20B and Cal-27 cells)	90
PLGA-PEG nanoparticle	Poly (lactic-co-glycolic acid)-PEG, Doxorubicin, R848, CCL20, Poly (I: C)	TC-1 tumor-bearing mice	91
Sterically stabilized cationic liposome	DC-Chol: DOPE: PEG-PE (4: 6: 0.06), CpG-ODN	Mouse HNSCC (KCCT873 cells)	92
PC7A nanoparticle	2-(Hexamethyleneimino) ethyl methacrylate (C7A-MA) monomer, PEG-b-PC7A copolymer, HPV-16 E7 peptide	TC-1 tumor-bearing mice	93
**Nanovaccine**			
Poly (propylene sulfide) nanoparticle	Poly (propylene sulfide) nanoparticle with disulfide conjugated peptide, HPV-16 E7 peptide, CpG-B 1826 oligonucleotide	TC-1 tumor-bearing mice	94
R-DOTAP cationic lipid nanoparticle	R-1,2-dioleoyl-3-trimethyl-ammonium-propane (R-DOTAP), HPV-16 E7 and other antigens	TC-1 tumor-bearing mice	67
DOTMA/DOPE liposome	DOTMA, DOPE, HPV-16 E7 mRNA	TC-1 tumor-bearing mice	60
PLGA nanoparticles	Poly (lactic-co-glycolic acid), HPV-16 E7 peptide, ploy (I: C)	TC-1 tumor-bearing mice	95
PLGA nanoparticles	Poly (lactic-co-glycolic acid), Mutated HPV-16 E7 and E6/E7 protein, R848, poly (I: C)	TC-1 tumor-bearing mice, cynomolgus monkey	96
DOTMA/DOPE liposome	DOTMA, DOPE, Plasmid encoding HPV-16 E6, E7	TC-1 tumor-bearing mice	69
Synthetic high-density lipoprotein nanodisc	1,2-Dimyristoyl-sn-glycero-3-phosphocholine (DMPC), ApoA1 mimetic peptide 22A, HPV-16 E7 peptide, MPLA, CpG	TC-1 tumor-bearing mice	97
Liposome	Cholesterol/DOPC/PEG-DSPE/maleimide-PEG-DSPE at 35/60/2.5/2.5 or 35/62.5/0/2.5 mol%, Anti-CD137, IL-2	TC-1 tumor-bearing mice	98
Mesoporous silica micro-rod	Mesoporous silica micro-rod, Polyethyleneimine, HPV-16 E7 peptide, GM-CSF, CPG-ODN	TC-1 tumor-bearing mice	99
Q11 peptide assembled nanofiber	Peptides Q11 (Ac-QQKFQFQFEQQ-Am), E7 (44-62) was appended to the N terminus of peptide Q11 through a flexible linker, Ser-Gly-Ser-Gly, HPV-16 E7 peptide	TC-1 tumor-bearing mice	100
Hyaluronic acid-modified cationic lipid-PLGA hybrid nanoparticles	Cationic lipid (3β-[N-(N′,N′, dimethylaminoethane)-carbamoyl] cholesterol hydrochloride, DC-Chol), Hyaluronic acid, poly (lactic-co-glycolic acid), HPV-16 E7 peptide, ploy (I: C), CpG-ODN	TC-1 tumor-bearing mice	101
Heterocyclic lipid nanoparticle	Dihydroimidazole-linked lipids A2-Iso5-2DC18 and A12-Iso5-2DC18, HPV-16 E7 mRNA	TC-1 tumor-bearing mice	102
Supercharged green fluorescent protein	Supercharged green fluorescent protein (+36 GFP), HPV-16 E7 DNA, protein	TC-1 tumor-bearing mice	103
PLGA nanoparticle	PLGA NP coated with murine aCD40-mAb FGK45, HPV-16 E7, Pam3CSK4, Poly (I: C), aCD40-mAb	TC-1 tumor-bearing mice	104
Carboxymethyl dextran-based polymeric conjugate	Carboxymethyl dextran, ovalbumin was chemically affixed to CMD via reductive amination between the reducing end group of CMD and the amino group of OVA, Ovalbumin peptide	TC-1 tumor-bearing mice	105
HIV tat peptide	18-mer cationic peptide RKKRRQRRRRAHYNEVTF (Tat-E7), HPV-16 E7 peptide, GM-CSF DNA	TC-1 tumor-bearing mice	106
PEG-PE micelle	Polyethylene glycol-phosphatidylethanolamine (PEG-PE) micelles, Ovalbumin peptide	TC-1 tumor-bearing mice	107
Bacterial outer membrane vesicles	Escherichia coli recombinant DH5α cell-derived outer membrane vesicles, HPV-16 E7 protein	TC-1 tumor-bearing mice	108
PLGA nanoparticles	Poly (lactic-co-glycolic acid), Cell or tumor lysate	HNSCC cell line (FaDu and FAT cells)	109
Tumor-derived autophagosome	Autophagosome secreted by SCC7 tumor cells	Mouse HNSCC (SCC7 cells)	110
Nanosatellite	Polysiloxane-containing polymer-coated iron oxide core with inert gold satellites, E6/E7 peptide +cGAMP	Mouse HNSCC (PCI-13, UMSCC22b, UMSCC47, and FaDu cells)	65
Liposome	Cationic lipid reagent DOTAP, Total tumor RNA	Nasopharyngeal Carcinoma cell line (C15 and C666-1 cells)	111
Branched amphiphilic peptide capsules	Peptides bis (FLIVIGSII)-K-K4 and bis (FLIVI)-K-K4, Plasmid DNA encoding HPV-16 E7	TC-1 tumor-bearing mice	112
Virus-like particles	E7 inserted into Hepatitis B virus core antigen (aa. 1-149), HPV-16 E7 epitope	TC-1 tumor-bearing mice	113
Liposome	DOTAP and cholesterol (1: 1 or 1: 0 molar ratio), Plasmid DNA, HPV-16 E7 peptide	TC-1 tumor-bearing mice	114
Chitosan-coated selenium nanoparticle	Chitosan-coated selenium nanoparticle, F*luc*-mRNA	Nasopharyngeal carcinoma cell line (KB cells)	115
PMIDA coated CoO nanoparticles	N-Phosnomethyliminodiacetic acid coated cobalt oxide nanoparticle, Human oral carcinoma (KB) cell lysate	Oral cancer cell line (KB cells)	116
Mesoporous silica rods	Mesoporous silica rods, GM-CSF + CpG-ODN + E7 peptide	Mouse HNSCC (MOC2-E6E7 cells)	117

Abbreviation: DBP-Hf: di (p-benzoato) porphyrin-hafnium; nMOF: nanoscale metal-organic framework; HNSCC: head and neck squamous cell carcinoma; CPTEG: 1,8-bis (p-carboxyphenox-y)-3,6-dioxaoctane; CPH: 1,6-bis (p-carboxyphenoxy) hexane; R848: dual TLR7/8 activator Resiquimod; PLGA: poly (lactic-co-glycolic acid); CCL20: Macrophage Inflammatory Protein-3 alpha (MIP3α); CpG-ODN: CpG Oligodeoxynucleotides; HPV-16: human papillomavirus type 16; TLR7/8: toll-like receptor 7 and 8; OSCC: oral squamous cell carcinoma; R-DOTAP: R-1,2-dioleoyl-3-trimethyl-ammonium-propane; DMPC: 1,2-Dimyristoyl-sn-glycero-3-phosphocholine; DOPC: Dioleoylphosphocholine; PEG: polyethylene glycol; DSPE: distearoylphosphoethanolamine; GM-CSF: granulocyte-macrophage colony-stimulating factor; Q11: Ac-QQKFQFQFEQQ-Am; GFP: green fluorescent protein; NP: nanoparticles; CMD: carboxymethyl dextran; OVA: ovalbumin; cGAMP: cyclic GMP-AMP; PMIDA: N-Phosnomethyliminodiacetic acid; CoO: cobalt oxide.
